# Relationship between BMI ≥25 and periodontal status: A case‒ control study

**DOI:** 10.15171/japid.2018.014

**Published:** 2018-12-25

**Authors:** Seyed Ali Banihashemrad, Kazem Fatemi, Taher Pakdel, Nahid Nasrabadi

**Affiliations:** ^1^Department of Periodontology, Faculty of Dentistry, Mashhad University of Medical Sciences, Mashhad, Iran; ^2^Private Practice, Mashhad, Iran

**Keywords:** Body mass index, clinical attachment loss, obesity, periodontal pocket depth

## Abstract

**Background:**

Obesity is an important subject in both developed and developing countries. Obesity is a risk factor for many diseases, including cardiovascular diseases, hypertension and osteoarthritis. Periodontitis is a prevalent, chronic disease and multiple factors have been proposed to contribute to its progression. we aimed to compare the periodontal status of normalweight and obese individuals.

**Methods:**

In this study, we consecutively selected 100 patients (50 obese and overweight as the case group, based on body mass index [BMI], and 50 others with normal weight, as the control group) referred to the Periodontology Department of Mashhad Dental School. The demographic data of the participants were recorded, including age, gender, height and weight. The following periodontal parameters were assessed: periodontal pocket depth (PPD), clinical attachment level (CAL) and plaque index. Kolmogorov-Smirnov test, chi-squared test and independent t-test, as well as ANCOVA, were used to analyze data.

**Results:**

We found that the mean PPD was similar in the test and control groups (P=0.168). Moreover, CAL was not significantly different between the two groups (P=0.494).

**Conclusion:**

Our findings indicated that obesity and overweight do not seem to have an association with periodontal parameters such as periodontal pocket depth and clinical attachment loss. Further research is needed to evaluate this relationship.

## Introduction

The prevalence of obesity and overweight has been on the increase universally during the past decades.^1^ This is a multifactorial condition due to extra storage of fat, arising from interaction of behavioral, cultural, psychological and genetic factors.^2^ On the other hand, obesity is a significant risk factor for various chronic conditions such as hypertension, type 2 diabetes, dyslipidemia, and cerebrovascular and cardiovascular diseases.^3^

Periodontitis refers to the destruction of periodontal attachment apparatus. It is one of the major and common oral health problems in the developing and developed nations.^4,5^

Although periodontal disease is triggered by special bacterial plaque, it is modulated indirectly by inflammatory cytokines that affect the periodontium and promote inflammation and bone destruction.^6,7^

Todays, it is clear that adipose tissue is a metabolically active and complex endocrine organ, which secrets numerous modulatory factors like tumor necrosis factor-α (TNF-α) and interleukin-6.^8^ These cytokines might have undesired effects on periodontium.^9^

The association between obesity and periodontal disease was first suggested by Perlstein et al in 1977. They reported more bone loss in obese rats than in normal ones.^10^

Some studies have reported that obese patients show high plasma concentration of proinflammatory markers. The fatty tissue secrets high quantities of TNF-α, which could suggest a relationship between periodontal disease and obesity.^11^ On the other hand, Kongstad et al^12^ showed that BMI might be inversely associated with clinical attachment loss. Moreover, Buduneli et al^13^ reported that obesity does not have a significant effect on periodontal parameters. Based on some controversial results from previous studies, we tried to examine the association between obesity and periodontal diseases in an Iranian adult population by using body mass index as measures of total body fat.

## Methods

This cross-sectionalstudy was conducted on 100 patients, consisting of 49 women and 51 men, with an age range of 19‒45 years (50 obese and overweight subjects with a mean age of 36.4±6.7 years and 50 normal subjects with a mean age of 28±3.8 years) referred to the Department of Periodontology, Mashhad Faculty of Dentistry. Informed consent was obtained from all the volunteer patients. The protocol of the study was approved by the Ethics Committee of the university.

Patients enrolled in the study were nonsmokers, without any history of systemic conditions such as diabetes, hypertension and pregnancy. None of them had taken antibiotics over the past three months.

Demographic data, including age, sex, weight and height were achieved by filling a questionnaire by the patients and based on calculated BMI for each subject, the patients were assigned to two groups. The rest of the indices were recorded by the same trained clinician throughout dental examinations. Periodontal indices included clinical attachment level (CAL) measured as the distance from the CEJ to the base of the probable pocket depth at six sites for each tooth (mesiobuccal, mid-buccal, distobuccal, distolingual, mid-lingual and mesiolingual) using a Williams periodontal probe and probing pocket depth (PPD) measured from the gingival margin to the base of pocket recorded at six sites mentioned above for all the teeth, using Williams Probe. Presence of plaque was assessed following Sillness and Loe plaque index (PI).^14^ BMI was used as an indicator of obesity in terms of height and weight. It was calculated from body mass in kilograms divided by the square of the body height in meters and then categorized as underweight (<18.5 $\frac{kg}{m2}$ ), normal weight (18.5‒24.9 $\frac{kg}{m2}$ ), overweight (25‒29.9 $\frac{kg}{m2}$ ) and obese (>30 $\frac{kg}{m2}$ ).^15^

Since there was no subject in our study with BMI<18.5, the volunteers were divided into normal (BMI<25) and obese (BMI≥25) groups. On the other hand, the obese group in our study consisted of both overweight and obese subjects and the normal group consisted of normal and underweight patients.

### Statistical Analysis

Data were analyzed with Kolmogorov-Smirnov test, chi-squared test, independent t-test and ANCOVA using SPSS 16. Statistical significance was set at P=0.05.

## Results

A total of 100 patients (51 males and 49 females) were recruited in the present study, with a mean age of 32.58±6.72 years and an age range of 19‒45 years.

Mean of BMI in the normal group was 23.22±1.36 (19.99‒24.99), with 30.35±2.57 (25.38‒38.49) in the obese group. Since we were going to compare normal and obese individuals together, two groups were defined in our study.

Chi-squared test was used to compare gender distribution between normal and obese groups; the results showed no statistical difference according to Table 1.

**Table 1:** Gender distribution in the case and control groups

		**Sex**		**Total** **Number (%)**
		**Male** **Number (%)**	**Female** **Number (%)**	
**Group**	**Normal**	22 (44)	28 (56)	50 (100)
	**Obese**	29 (58)	21 (42)	50 (100)
**Total**		51 (51)	49 (49)	100 (100)
**Chi-squared test**		P=0.161		

Means of age and PI variables were significantly higher in the obese group (Table 2).

**Table 2:** Comparison of age and PI in the case and control groups (N=50)

**Variable**	**Group**	**Mean ± SD**	**Range** **(min max)**	**P-value**
**Age**	**Normal**	28.74±3.89	(19 37)	<0.001
	**Obese**	36.42±6.79	(25 45)	
**PI**	**Normal**	1.03±0.27	(0.15 2.20)	<0.001
	**Obese**	2.04±0.48	(1.20 3.20)	

**PI:** plaque index

Hence, ANCOVA was used to compare PPD and CAL by controlling the effect of age and PI. The results showed that the scores of the three variables exhibited greater increases in the case group but the difference was not significant (Table 3).

**Table 3:** Comparison of periodontal indices between the control and case groups by ANCOVA

**Variable**	**Group**	**Number**	**Mean ± S.D**	**Range** **(min max)**	**P-value**
**PPD**	**Normal**	50	1.58± 0.26	(1 2.5)	0.0168
	**Obese**	50	2.39±0.50	(1.5 3.5)	
**CAL**	**Normal**	50	1.33±0.35	(1 2)	0.494
	**Obese**	50	2.00±0.49	(1.2 3.5)	

**PPD:** periodontal pocket depth; CAL: clinical attachment level

## Discussion

Recent studies have proposed that overweight and obesity are a risk factors for initiation and progression of periodontal diseases.^9-12^ It seems obesity involves immune‒inflammatory responses which increase susceptibility to periodontitis.^16^

Lundin et al^17,18^ noted a relation between TNF-α in the gingival crevice fluid and BMI.

The results of the present study are in contrast to some previous reports, which suggest a relationship between obesity and periodontal diseases. For example, Saito’s study on Japanese adults^19^ showed that an increase in BMI was associated with an increase in the risk of periodontitis. Al-Zahrani et al^20^ reported a significant association between the body fat and periodontitis only in younger adults, not middle-aged or older adults. Sarlati et al^15^ noted a correlation between BMI and various periodontal measures, including pocket depth, clinical attachment loss and plaque index in a population of young Iranian adults. Dalla Vecchia et al^21^ suggested that obesity was significantly associated with periodontal diseases in adult, nonsmoking women.

With regard to all the above, we decided to select a rather small sample size of middle-aged Iranian adults to evaluate this relationship. We selected the volunteers in our study from patients who referred to Mashhad Dental Faculty, which is the biggest center in eastern Iran. Because of unique status (cost and quality of services), we could make sure that the volunteers in our research were a sample of Iranian society. To the best of our knowledge, there is no similar study in Iran to compare periodontal parameters in obese and normal weight subjects. In the present study, we used body mass index as measures of total body fat. After analyzing data, no association was observed between obesity and periodontitis. The lack of association was confirmed after adjusting for plausible confounding variables (age and plaque accumulation). These two items are assumed important to develop periodontitis. First, the results showed a positive association between poor periodontal status and overweight among adults. Therefore, after using ANCOVA to control confounding variables, a significant relationship was not found. Since plaque accumulation increases the chances of periodontitis, it seems the effect of plaque accumulation on periodontium is more influential than obesity. Therefore, failure to match the samples was one of the limitations of our study.

The results of the present study are consistent with a study conducted by de Castilhos et al,^22^ who reported no association between periodontitis (measured by pocket depth) and obesity (measured by waist circumference) among 720 young people. According to Awad et al^22,23^ and de Castilhos et al^22^ periodontitis might have been underestimated by using PPD, so that we assessed the periodontal disease by CAL.

The two studies above reported that the probable reason for lack of association between periodontitis and obesity was age of the participants who were young (about 20 to 30 years old). The mean age in our study was 32 years, which is higher than those studies.

The limitation of our study was its cross-sectional design. However, the gender of cases and control subjects was similar, was no significant difference between the normal and obese groups. We did not evaluate factors influencing life style such as oral hygiene habits, emotional status, diet and other items. However, the effect of these variables may have been possible.

## Conclusion

In view of these findings, there was no association between obesity and periodontal disease in the subjects in the present study. Larger sample sizes and longitudinal clinical trials are necessary to determine a causal relationship between them.

## Authors’ contributions

SABR proposed the idea and collected data. KF collected data and prepared the manuscript. TP collected data and prepared the manuscript. NN prepared the manuscript and revised it.

## Competing interest

Authors declare no conflict of interests.

## Ethics approval

The protocol of the study was approved by Ethics Committee of the University (code number: IR.MUMS.REC.1387.900)
